# Large Language Models in Cancer Imaging: Applications and Future Perspectives

**DOI:** 10.3390/jcm14103285

**Published:** 2025-05-08

**Authors:** Mickael Tordjman, Ian Bolger, Murat Yuce, Francisco Restrepo, Zelong Liu, Laurent Dercle, Jeremy McGale, Anis L. Meribout, Mira M. Liu, Arnaud Beddok, Hao-Chih Lee, Scott Rohren, Ryan Yu, Xueyan Mei, Bachir Taouli

**Affiliations:** 1 Biomedical Engineering & Imaging Institute, Mount Sinai Health System, New York, NY 10029, USA; 2 Department of Diagnostic, Molecular and Interventional Radiology, Mount Sinai Health System, New York, NY 10029, USA; 3 Department of Radiology, Columbia University Irving Medical Center, New York, NY 10032, USA; 4 Department of Radiation Oncology, Institut Godinot, 51454 Reims, France; 5 Faculty of Medicine, Université de Reims Champagne-Ardenne, CRESTIC, 51100 Reims, France; 6 Yale PET Center, Department of Radiology & Biomedical Imaging, Yale University School of Medicine, New Haven, CT 06520, USA

**Keywords:** large language model, artificial intelligence, imaging, cancer

## Abstract

Recently, there has been tremendous interest on the use of large language models (LLMs) in radiology. LLMs have been employed for various applications in cancer imaging, including improving reporting speed and accuracy via generation of standardized reports, automating the classification and staging of abnormal findings in reports, incorporating appropriate guidelines, and calculating individualized risk scores. Another use of LLMs is their ability to improve patient comprehension of imaging reports with simplification of the medical terms and possible translations to multiple languages. Additional future applications of LLMs include multidisciplinary tumor board standardizations, aiding patient management, and preventing and predicting adverse events (contrast allergies, MRI contraindications) and cancer imaging research. However, limitations such as hallucinations and variable performances could present obstacles to widespread clinical implementation. Herein, we present a review of the current and future applications of LLMs in cancer imaging, as well as pitfalls and limitations.

## 1. Introduction

Large language models (LLMs) [such as ChatGPT (OpenAI), Llama (Meta), Gemini (Google) and more recently DeepSeek (DeepSeek)] are artificial intelligence (AI)-based text generators that have recently seen exponential growth in the medical field [1,2]. In radiology, LLMs have been investigated as a tool to help overcome challenges encountered by oncologists and radiologists in cancer imaging [3,4]. More specifically, LLMs can assist physicians and patients by improving the standardization and quality of imaging reporting for cancer patients, as well as contribute to individual prognostication and risk-assessment. They can also be utilized in translating complex medical information into more accessible, patient-friendly language [5,6]. The aim of this review is to demonstrate the current applications of LLMs in cancer imaging by providing an overview of the technical principles of LLMs, covering current applications and possible future areas of innovation, as well as highlighting limitations of their use when applied to cancer imaging.

This scoping review includes articles discussing the use of LLMs for medical imaging and oncology and focuses on cancer imaging. These were identified in PubMed, Web of Science, and Scopus (using the keywords “Large Language Models”, “cancer”, “imaging”, “oncology” in the title or abstract). Articles published in other languages than English were excluded.

## 2. Large Language Models: Definitions and Different Architectures

### 2.1. LLM Definition

Large language models (LLMs) are a family of machine learning (ML) models that consist of more than a billion parameters and are pre-trained on massive text datasets to process and learn from natural language [1]. These models are typically transformer-based neural network algorithms built around the attention mechanism as their building blocks (Table 1). The attention mechanism addresses limitations of earlier models, such as recurrent neural networks (RNNs), in capturing long-range dependencies, allowing models to focus on the most relevant parts of the input text. Researchers have observed that the performance of transformer-based models in understanding natural language improves with the increase in model size, training data volume, and computational resources, a phenomenon known as “scaling laws”. As their capabilities have grown, pre-trained LLMs have demonstrated the ability to reason with minimal or even no training samples, setting them apart from traditional supervised learning models.

LLMs can be categorized based on their architectures, which generally fall into three types: encoder-only, decoder-only, and encoder–decoder models [2]. The encoder and decoder are the two primary components of the transformer model. Conceptually, the encoder maps the input text into a high-dimensional “embedding” space, where every input text is represented as a sequence of numerical vectors. The decoder uses embeddings and the input text to generate output sequences, such as translated text or autocompleted text, depending on the use cases.

### 2.2. Encoder-Only Models

As the name implies, this family of models uses only the encoder layers as building blocks. Encoder-only models are pre-trained using masked language modeling to learn contextual information between words in a corpus. Specifically, words in the texts are randomly removed, and the models are trained to predict these randomly masked words during pretraining.

Masked language modeling forces the model to predict missing words based on their context, thereby capturing relationships between words and their surrounding context. A pre-trained model can then be fine-tuned for language understanding tasks such as sentiment classification, question answering, and entity recognition. BERT, RoBERTa, ERNIE, and ALBERT are representative examples of encoder-only models [7,8,9].

### 2.3. Decoder-Only Models

Decoder-only models rely exclusively on decoder layers as their foundational components. These models are typically trained using autoregressive modeling rather than masked language modeling. In this approach, the model learns to predict the next word or token based on the preceding context. GPT-1 was the pioneering model to demonstrate that a decoder-only architecture could excel across a wide range of natural language tasks, sparking the further development of decoder-only models. ChatGPT (by OpenAI), DeepSeek (by DeepSeek) and LLaMA (by Meta) are all examples of decoder-only models [10,11].

### 2.4. Encoder–Decoder Models (Figure 1)

Encoder–decoder models, such as T5 and BART, were developed to transform a sequence into another sequence (sequence-to-sequence modeling). This architecture makes them particularly well-suited for tasks where the output strongly depends on the input, such as text summarization, translation, and answering questions.

**Figure 1:**
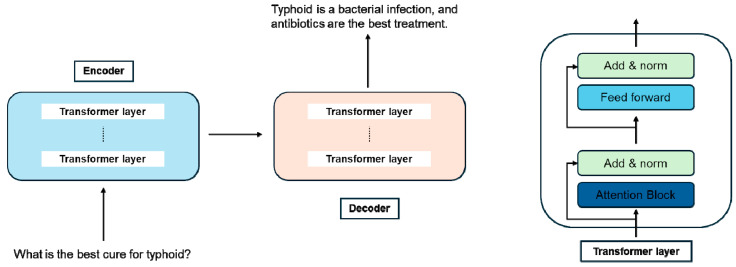
The architecture of a general LLM encoder–decoder framework. The input is encoded into tokens via the encoder for the decoder to generate the output. Both encoder and decoder modules were built by stacks of transformer layers.

## 3. Current Challenges in Cancer Imaging

**Figure 2:**
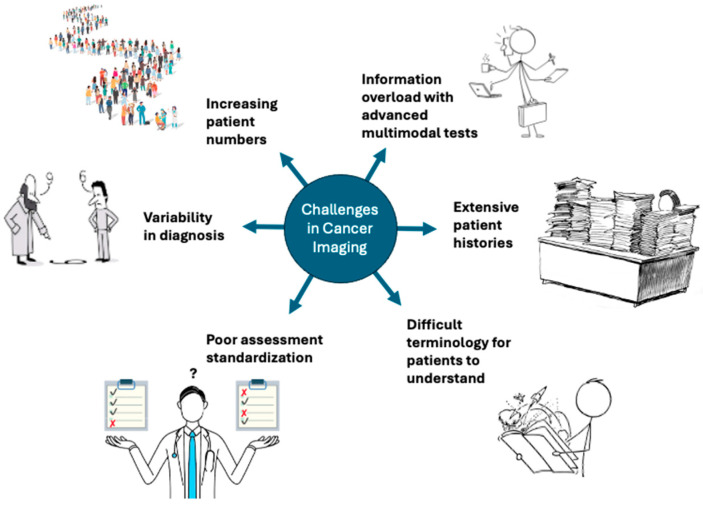
Despite advances in radiology, challenges in cancer imaging remain on both the physician side and the patient side. This includes an ever-increasing patient load, information overload from newer modalities and techniques, extensive relevant patient histories, poor standardization of assessment strategies, variability in diagnoses, and difficulty in patient communication and understanding. LLMs are one potential way of supporting radiologists in these challenges.

There are numerous global challenges in cancer imaging surrounding the complexities of cancer diagnosis and inter-patient and inter-physician variability (Figure 2). Limitations of standardization in utilizing precision imaging for early detection and treatment planning limits globalization [12]. According to the National Cancer Institute (NCI) and the International Agency for Research on Cancer (IARC), there were near 20 million new cancer cases worldwide in 2022, with a predicted increase to 29.9 million by 2040 [13]. This increasing incidence paired with stagnant growth in the number of practicing radiologists has created a clear resource mismatch [14,15]. Novel national screening strategies such as lung cancer screening may increase this burden [16]. In addition, advancements in imaging protocols, increases in watch-and-wait strategies, and more favorable outcomes with novel treatments lead to larger and more complex information that must be integrated into a clinical report.

The burden on radiologists could be reduced by technologies such as LLMs and other AI and ML models that can bolster diagnostic performance, reduce variability, and improve overall efficiency. AI models could help streamline standardization procedures for oncologic imaging assessment, such as providing standardized summary reports, assisting prediction based on clinical reports, and assisting in RECIST classification for cancer response [17]. Further, they could provide support for radiologists with the automatic fusion of clinical information, genomics, radiomics, and multimodal imaging to provide a more comprehensive understanding of disease while reducing information overload [18]. Lastly, patients are often not familiar with image interpretation nor have the knowledge needed to understand and interpret complex clinical reports. LLMs may also help on the patient side by producing lay language summaries and analyses to help patients understand their own health information without adding to the burden on radiologists.

## 4. LLMs for Radiology Exam Protocol Standardization

Radiologists play a crucial role in protocoling radiology exams in a cancer setting, which involves reviewing patients’ clinical information on the electronic health record (EHR) and prescribing the most appropriate set of imaging services. Traditionally, protocoling is performed by radiologists, residents/fellows, or technicians to tailor the studies to the patient’s clinical needs, but this process can be time-consuming and is susceptible to inter-operator variability.

LLMs have demonstrated promise in reducing variability and increasing the speed of protocoling [19]. In one instance, an emergency radiology department employed Chat-GPT 4 to select the appropriate protocol for an imaging study [20]. Two radiologists evaluated the performance and found that Chat-GPT was able to select the clinically relevant study with an average score of 4.5 out of 5. In another study, brain MRI protocolization was automated using an LLM classifier. The authors found that the LLM was able to automatically classify roughly two-thirds of cases with 95% accuracy [21]. For the other one-third of cases that the LLM flagged for manual review, it still selected the appropriate protocol in 92% of cases.

Although there is a lot of promise in LLMs to assist radiologists and staff with protocoling, it is not without its challenges. One challenge is the intrinsic bias in the training data, which leads to varied responses in different practice settings [22]. Another concern is the translation of LLM protocols to clinical performance. For instance, when limited clinical information is available, a well-trained radiologist or staff will often reach out to the related team. It is yet to be seen how LLMs will navigate these crucial exam settings, and what the realized clinical outcomes will be for their protocolization. A final concern is data security. With clinical information being fed through LLMs, there are risks of data breaches which may expose patient data to an external server that is not properly protected, leaving them at risk. Without proper measures, improper security could lead to a breach of patient privacy, and cybersecurity threats could potentially compromise suggested protocols and procedures, leading to patient harm [23].

With these challenges in mind, there is ongoing work to improve LLMs’ ability to assist radiologists, such as training LLMs to extract relevant information from the EHR. As research progresses, it will be crucial to address these challenges and establish robust frameworks for integrating LLMs into radiology workflow, ensuring they serve as effective complementary tools.

## 5. LLMs for Improving Reporting

The applications of LLMs in cancer imaging reporting are vast, spanning a wide range of tasks. These include the standardization of imaging reports with automated classifications, as well as the identification of speech recognition errors, language correction, and medical text summarization and simplification.

First, the generation of structured reports represents a highly useful application of LLMs, contributing to the standardization and organization of medical content, thereby assisting radiologists in creating reports. For instance, one study highlighted GPT-4’s 100% accuracy in automatically aligning MRI/CT reports of various body regions with the corresponding report templates, converting these reports into JavaScript Object Notation (JSON) format, and structuring them without errors, loss of precision, or the introduction of additional findings [24]. The ability of LLMs to generate text from prompts can be extended to cancer imaging reports which usually includes demarcated sections for “Indication”, “Findings” and “Impression”. The LLMs can either improve or verify a currently existing report [25] or help generate expert level reports. Several models were recently trained to generate “Impression” based on the “Findings” section of imaging reports [26,27], with the final report evaluated using different metrics to assess the quality and accuracy of the text [28]. Additionally, LLMs have the potential to label imaging reports [29] and to flag urgent issues which need specific attention (e.g., a pulmonary embolism in the setting of a oncological thoracic abdominopelvic CT-scan).

Furthermore, LLMs can transform unstructured CT scan reports into structured formats. Chat GPT-4 effectively converted free-text CT reports for head and neck carcinoma into standardized reporting templates [30]. An additional potential use of LLMs is improving references to prior reports, narrowing down differential diagnoses and supporting clinician decision making. The combination of a retrieval-augmented generation (RAG) LLM system with an extensive database of previous PET imaging reports from patients with breast cancer, lung cancer, and lymphoma enabled the identification of similar cases and the extraction of potential diagnoses based on those cases [31].

LLMs can be leveraged to improve the accuracy of voice-to-text transcription in report generation by identifying errors in speech recognition. GPT-4 has demonstrated very good performance, with an F1 score of 86.9% for detecting clinically relevant errors and 94.3% for non-clinically significant errors in radiology reports. It has proven effective in identifying internal inconsistencies and nonsensical errors [32]. New studies indicate a promising role for LLMs, not as substitutes for clinicians, but as tools to alleviate the documentation burden, thereby enabling clinicians to focus more on patient care [33].

## 6. LLM-Based Cancer Classification and Staging

Cancer staging generally enables the evaluation of disease extent and progression, as well as the personalization of treatment plans. Recent advancements in LLMs allowing for the automated interpretation of medical data including imaging reports have led to more accurate cancer staging. LLMs offer substantial potential to enhance tumor, node, and metastasis (TNM) classification, particularly in complex cancer staging, thereby supporting clinical decision making. A recent study evaluated NotebookLM, a retrieval-augmented generation LLM for lung cancer staging based on CT findings and showed promising results with better performance than GPT-4o [34]. The incorporation of cancer-specific features can significantly improve LLM accuracy in staging cancer lesions and may be effectively adapted to a broad spectrum of cancer types. Moreover, variations in performance and accuracy are observed, with some LLMs outperforming others in specific applications. As an example, GPT-4o has consistently shown higher overall lung cancer staging accuracy across multiple studies when compared to earlier models such as GPT-3.5 and GTP-4 [35]. Similarly, LLMs showed good performances for Breast Imaging Reporting and Data System (BI-RADS) classification based on text-based assessments but more limited ones for visual diagnosis [36]. A model to classify thyroid nodules with the ACR Thyroid Imaging-Reporting and Data System (TI-RADS) based on the features of these nodules in the ultrasound reports demonstrated an accuracy of 0.84 [37]. Another study demonstrated the more limited capabilities of LLMs to assign Prostate Imaging-Reporting and Data System (PI-RADS) categories based on MRI text reports from older models such as GPT-3.5 when compared to radiologists. While more recent models have shown overall improved accuracy for PI-RADS classification, performances are still variable across datasets, highlighting that improvement in the current LLMs is needed before they can be used in clinical practice [38]. Hybrid applications combining LLMs with deterministic elements may improve the performances of these models, such as in a recent study evaluating GPT-4 for O-RADS MRI scoring [39].

Response Evaluation Criteria in Solid Tumors (RECIST) 1.1 is a standardized way to assess therapy-related changes in cancer lesions with imaging. Response evaluation presents challenges including variability in expertise level, adherence to guidelines, and the use and access to individual contextual patient information. Some of this discordance could be mitigated through the adjudication or use of multiple readers, but variability persists. ML and LLMs could simplify this discordance by providing contextual patient background information to readers upon RECIST assessment as well as summaries of previous reports. Use of this information could reduce variability in reader interpretations and decrease diagnostic uncertainty [40].

Widespread variability in RECIST responses also exists due to the different selection of lesions between radiologists, diminishing the reproducibility of RECIST assessments [41]. In a study conducted by Bucho et al. [42], ML models were compared to radiologists in the selection of measurable and target lesions for RECIST assessment. The models mirrored the selection of lesions by the radiologists utilizing size and rank as primary selection parameters. The study showed significant inter-reader variability and furthered the importance placed on the implementation of more detailed standardization processes for lesion selection for RECIST assessment to ultimately lessen the dependence on interpretation at the individual radiologist level [42].

## 7. LLM for Individual Prognostication

In addition to applications in cancer classification and staging, recent advances in LLMs have also shown increasing potential in cancer status monitoring and prognostication through the integration of multimodal data such as imaging, clinical reports, and pathology findings. Arya et al. [43] employed a fine-tuned version of Google’s off-the-shelf Bi-directional Encoder Representations from Transformers (BERT) model to analyze radiology reports (MRI, CT, PET, etc.), achieving high accuracy in identifying cancer status and streamlining data curation for prognostication tasks. Despite the model’s promising performance, it was trained on a single-center dataset, raising questions about its generalizability. Moreover, only one LLM architecture was evaluated, so the field is still ripe for exploration using improved techniques such as the RAG models. Kim et al. [44] proposed a multi-modal approach including the LLM-guided integration of CT images, pathology slides, and clinical data, demonstrating high precision and recall for the prediction of 5-year survival in lung cancer. By implementing a novel multimodal alignment module (MAM) and a custom feature aggregator, their model outperformed previous multimodal architectures [45,46] where features were simply concatenated and aggregated through a fully connected layer (prediction AUCs of ~0.85 and ~0.67, respectively). However, LLM guidance relied on manually generated prompts, potentially compromising the performance of a model which is highly sensitive to prompt quality. Similarly, a study by Kim et al. [47] reported high accuracy for the prediction of overall survival in patients with pancreatic cancer using the Bidirectional Encoder Representations from Transformers (ClinicalBERT) model, trained on CT scan reports. Finally, Tay et al. [48] reported high F1 scores from various LLMs in the prediction of metastatic sites for several primary cancers using CT, PET-CT, and MRI reports. Both studies [41,42] were based on clinical reports from a limited number of centers, again underscoring the need for multi-center, prospective validation. For the accurate prediction of metastatic sites, Bucho et al. [42] also acknowledged the need for training datasets tailored to each specific cancer type, e.g., cancers where primary tumors may metastasize to rarer sites like the pancreas, spleen, thyroid, and ureter.

Other contributions in the field have emphasized the use of LLMs in the prediction of treatment outcomes for various cancer types. Tan et al. [49] reported the high accuracy of a prompt-based fine-tuned GatorTron model in the classification of treatment response for colorectal, lung, breast, and gynecological cancers from CT scan reports. In a more recent study by Xiang et al. [50], the Multimodal transformer with a Unified maSKed modeling (MUSK) model, pretrained on unlabeled, unpaired pathology image and text data, showed excellent performance in tasks such as melanoma relapse prediction and immunotherapy response prediction in lung and gastro-esophageal cancers.

As noted, several works cited in this section relied on datasets from a few medical centers. This represents a selection bias in the populations included in model development and validation. The widespread clinical implementation of LLMs alone or in multimodal combinations requires multi-center prospective validation, where the main challenge is ensuring the integrity and privacy of patient information as it is shared among sites. Federated learning schemes, where parameters from locally trained models rather than patient data are shared among institutions, have been proposed as a potential solution to this obstacle [51].

LLMs also suffer from a general lack of contextual understanding. They rely solely on the data presented to them at training and may fail in more complex medical tasks for which they were not trained. Moreover, LLMs in isolation are by definition limited to text (clinical) data and, hence, cannot interpret visual information, like pathology or radiology images. This results in diagnoses or predictions based on limited data. However, some of the works cited above have begun to explore multimodal models combining LLMs, radiology, and pathology imaging to overcome some of the challenges of limited context and interpretation.

Despite these limitations, these promising early studies suggest that the future development of multimodality approaches and evaluation in multi-center studies could bring LLM-based models closer to clinical implementation for cancer prognostication and outcome prediction.

## 8. LLMs for Patient Communication

LLMs have significant potential to improve patient communication through education and accessibility. These models can work to provide a more personalized approach to medical care and increase access to medical information [52]. These LLMs present even further utilization in radiology for report summarization and simplification to enhance overall patient comprehension. In a study conducted with ChatGPT-4o for the simplification of breast imaging reports, 21 radiology reports were selected for simplification by a radiologist with 20 years of experience. Five radiologists specializing in breast imaging examined the quality of the simplified reports generated by ChatGPT and scored them using a 5-point Likert scale. The overall results showed high scores from the radiologists on the factual accuracy and completeness of the simplified reports. A good median score was provided for the potential harm category as well. Excellent median Likert scores for mammography and ultrasound were found, as well for the comprehension of the non-healthcare readers [53]. These results highlight the potential reliability of LLMs in simplifying imaging reports to strengthen patient communication. This can be taken a step further with the translation of imaging reports in multiple languages, decreasing the challenge of travelers or patients who speak different languages [54].

## 9. Pitfalls and Limitations

Despite advances in LLMs, several challenges impede widespread clinical deployment. Primarily, the LLMs are subject to hallucinations and may provide false information or answers. This is particularly problematic for healthcare information and cancer imaging, where hallucinations may be harmful. Improved methods of detection of these hallucinations are essential for the clinical implementations of these models [55]. A foremost challenge faced by these models is the extraction of relevant clinical data, especially in oncological patients with lengthy history, with sources ranging across the EHR from the study notes to the patient’s clinical chart. Another limitation with LLMs is their variable response, particularly in nuanced cases such as choosing correct radiologic procedures. If LLMs often provide different answers (including sometimes false information) when prompting the same question multiple times regarding cancer imaging, their implementation for clinical care is impossible. For example, in instances with high variability, LLMs struggle to perform. A study found that neuroradiologists significantly outperformed LLMs when deciding neuroradiological procedures due to the variance in responses produced by the LLMs [56]. LLMs also present several limitations for medical reporting, including issues with technical terminology, inaccuracies in interpreting medical data, incomplete information, and biases, such as those related to underrepresented racial groups in cardiovascular disease data [24]. Finally, LLMs may be limited in cancer classification and staging due to the requisite processing of information indiscriminately from diverse sources, occasionally generating answers based on unreliable, outdated references, or inadequate referencing [34].

## 10. Future Perspectives

### 10.1. Tumor Boards

As oncological decision making continues to become more complex, traditional multidisciplinary tumor boards (MTBs) often struggle with information overload and limited resources. Macchia et al. [57] illustrated how data-driven, AI-assisted solutions could help overcome these constraints in Locally Advanced Cervical Cancer (LACC) management, through the automatic classification of clinical stage and the flagging of discrepancies across diagnostic methods. Their proof-of-concept “Smart Virtual Assistant” demonstrated high staging accuracy (94–98%) and highlighted the most complex cases that warranted in-depth discussion. This early evidence underscores a critical need for computational tools capable of bridging knowledge gaps and streamlining the decision-making process in multidisciplinary environments.

Subsequent investigations point to similar applications and limitations in other tumor types, reinforcing the notion that LLMs can offer pragmatic, though not definitive, clinical support. Schmidl et al. [58] show how ChatGPT 3.5 and 4.0 can efficiently list treatment options for head and neck squamous cell carcinoma but sometimes propose interventions outside guideline recommendations. Zabaleta et al. [59] similarly found that ChatGPT, when supplemented with formal guidelines, can achieve up to 75% concordance with a thoracic MTB in non-small-cell lung cancer. Meanwhile, Sorin et al. [60] reported that ChatGPT-3.5’s recommendations align with final board decisions in 70% of ten breast cancer cases, indicating moderate promise. Across these studies, the importance of human oversight and individualized clinical insight remains paramount, with each group concluding that AI systems are best deployed as adjunctive tools rather than standalone decision makers.

Looking ahead, the successful integration of LLMs into tumor boards will likely involve the continued refinement of AI models, better alignment with evidence-based guidelines, and robust mechanisms for addressing context-specific nuances like patient comorbidities or preferences [61]. Prospective and multicenter trials are essential to validate both performance and safety, while ethical and regulatory frameworks must evolve to ensure transparency and accountability. Ultimately, these combined efforts can foster more efficient MTBs—supporting clinicians by synthesizing complex data, drawing attention to atypical or contradictory findings, and offering an educational platform for trainees—without supplanting the critical role of expert clinical judgment.

### 10.2. Preventing Adverse Events

Imaging in oncological settings is invaluable but also comes with a few risks. First, the number of rare but feared severe allergic reactions (“anaphylactic type”) to contrast agents and, in particular, iodine-based contrast materials, increase with the volume of enhanced CT-scans performed, which is the case for most of the initial workup and follow/up of patients with cancers. Risk factors for these reactions include previous allergic reactions, chronic illnesses, and atopic tendencies. A higher risk of allergic reactions could be helped by prescribing premedication drugs and switching to low-osmolarity contrast media [62,63]. Thus, early detection is essential and LLM could play a role. In theory, LLM-based automated questionnaires before contrast-enhanced examinations could detect patients at risk and flag them, enabling overall increased imaging safety. LLMs could play additional roles in identifying patients at risk for certain exams, including radiation-based imaging in pregnant patients or MRIs in those with contraindications such as pacemakers or other metallic implants.

### 10.3. Drug Development

LLMs have the potential to be transformative in oncology drug development by enhancing the identification and investigation of prognostic, predictive, and response biomarkers. The ability of these AI-driven models to systematically analyze vast datasets—ranging from radiology and pathology reports to genomic sequences and electronic health records—and generate clinically meaningful insights could lead to accelerated drug discovery and the implementation of more personalized treatment strategies.

### 10.4. Prognostication

LLMs can aid in identifying biomarkers that predict overall survival or disease progression independent of treatment. By integrating longitudinal imaging data, genomic alterations, and clinical outcomes, LLMs can stratify patients based on risk profiles, allowing for early interventions in high-risk groups. For example, LLMs can analyze imaging and pathology reports to infer tumor burden and liver metastasis patterns, which are linked to worse prognosis and reduced treatment efficacy across various cancers [64,65,66]. Additionally, LLMs can be applied to large-scale EHR-based data and have been able to accurately predict overall survival in lung cancer patients [67].

### 10.5. Predictive Value

Predictive biomarkers help clinicians identify which patients are most likely to benefit from a specific therapy. LLMs can assist in refining these biomarkers by correlating genomic data (e.g., PD-L1 expression, tumor mutational burden) with imaging-derived metrics and clinical response patterns. Additionally, LLMs can process vast multi-omics datasets—combining transcriptomics, proteomics, and metabolomics—to uncover novel resistance mechanisms that may inform combination therapy strategies [50]. For example, LLMs can analyze imaging and pathology reports to detect tumor metabolic volume and elevated spleen or bone marrow metabolism on 18F-FDG PET/CT, indicative of an overall immunosuppressive microenvironment and a lower likelihood of response to immunotherapy [68].

### 10.6. Response Assessment

Tracking intra-treatment tumor response with high accuracy is also critical to allow for rapid therapeutic adaptation. LLMs could facilitate the real-time evaluation of response biomarkers by integrating radiomic features to capture the earliest changes in tumor character. LLMs could also enhance and facilitate the extraction of data, including disease progression data from free-text EHRs, which would improve the accuracy, efficiency, and scalability of real-world evidence generation for lung cancer treatment response assessment [49]. LLMs could even be trained to automate all response classification tasks by systematically retrieving information as it is generated from clinical trials [69,70].

### 10.7. Treatment Selection

LLMs have also been used to enhance clinical decision support by integrating domain-specific medical data through the retrieval-augmented generation of clinically relevant, evidence-based oncology treatment recommendations with high concordance to expert suggestions [71].

## 11. Summary

**Figure 3:**
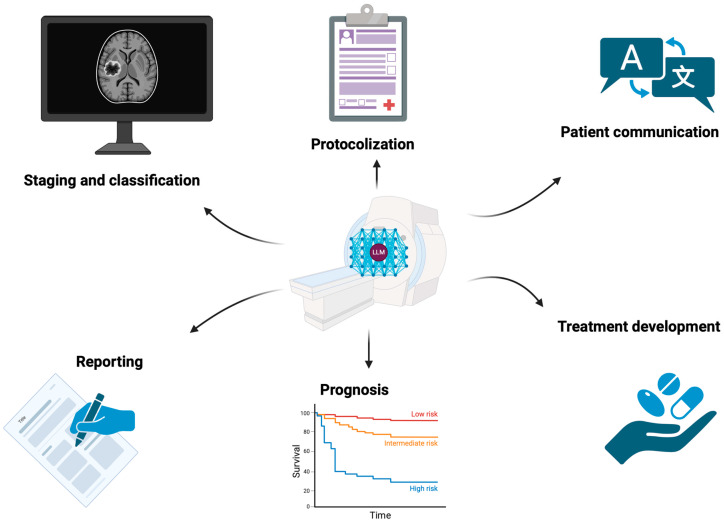
Current and future applications of large language models in cancer imaging. The Chinese Wén (文) character can be translated to mean “language” or “writing”. Figure created using BioRender, version 04.

This scoping review demonstrates the potential of LLMs for various purposes in cancer imaging and more globally at every step of patient care in oncology [3]. Different models, with various architectures and variable performances, have the potential to overcome many challenges in cancer imaging, such as faster and more standardized protocolization and reporting, and improved staging and prognostication, in addition to better radiologist/patient communication with an improved overall understanding of imaging reports (Figure 3). These models can be a part of daily care in the future and support not only adequate prevention and detection in imaging examinations but also future treatment developments. Their potential in cancer research will also continue to be expanded [72]. However, though LLM technology is promising, clinicians and researchers should acknowledge their current limitations and potential biases, especially in sensitive settings such as cancer imaging, since these models are vulnerable to data-poisoning attack and misinformation [73,74].

## Figures and Tables

**Table 1:** Architecture, availability and time of release of the different LLMs (including the ones with updates being released in 2025).

LLMs	Type	Availability	Time of Release
BERT	Encoder-only	Open-source	2018
RoBERTa	Encoder-only	Open-source	2019
ERNIE	Encoder-only	Open-source	2019
ALBERT	Encoder-only	Open-source	2019
GPT—series	Decoder-only	Closed-source	2018–Current
DeepSeek—series	Decoder-only	Open-source	2023–Current
LLaMA—series	Decoder-only	Open-source	2023–Current
T5	Encoder–Decoder	Open-source	2019–Current
BART	Encoder–Decoder	Open-source	2019
